# Optimizing Antiemetic Strategies Across Phases of Chemotherapy-Induced Nausea and Vomiting: Real-World Evidence in Breast Cancer

**DOI:** 10.3390/curroncol33020078

**Published:** 2026-01-28

**Authors:** Akif Doğan, Hande Nur Erölmez, Goncagül Akdağ, Sedat Yıldırım, Özlem Nuray Sever

**Affiliations:** 1 Department of Medical Oncology, University of Health Sciences, Sancaktepe Şehit İlhan Varank Training Research Hospital, Istanbul 34785, Turkey; ozlem.sever@hotmail.com; 2 Department of Family Medicine, University of Health Sciences, Sancaktepe Şehit İlhan Varank Training and Research Hospital, Istanbul 34785, Turkey; herolmez@gmail.com; 3 Department of Medical Oncology, University of Health Sciences, Kartal Lütfi Kırdar City Hospital, Istanbul 34865, Turkey; akdaggoncagul@gmail.com (G.A.); rezansedat@hotmail.com (S.Y.)

**Keywords:** chemotherapy-induced nausea and vomiting, highly emetogenic chemotherapy, neurokinin-1 receptor antagonists, aprepitant, fosaprepitant, breast cancer

## Abstract

Background: Chemotherapy-induced nausea and vomiting (CINV) remains one of the significant challenges in oncology despite guideline-based prophylaxis, particularly in patients receiving highly emetogenic chemotherapy (HEC). While neurokinin-1 (NK-1) receptor antagonists are established as a key component of standard antiemetic regimens, evidence of their phase-specific effectiveness in real-world, homogeneous patient populations remains limited. This study aimed to determine which antiemetic regimen provides optimal control in each CINV phase to support a tailored prophylactic approach. Methods: This single-center, retrospective, real-world study included 260 female patients with stage II–III breast cancer receiving anthracycline–cyclophosphamide-based HEC. All patients had similar demographic and clinical characteristics, forming a relatively homogeneous cohort. Each received a triple antiemetic regimen consisting of a 5-HT_3_ receptor antagonist, dexamethasone, and an NK-1 receptor antagonist (either a single-dose intravenous fosaprepitant or a 3-day oral aprepitant). Complete response (no vomiting and no rescue therapy) and no-vomiting rates were assessed in the acute (0–24 h), delayed (24–120 h), and overall (0–120 h) phases. Results: In this relatively homogeneous cohort of high-risk patients, fosaprepitant-based prophylaxis achieved better symptom control during the acute phase, whereas aprepitant-based regimens were more effective in the delayed and overall phases. These findings suggest phase-specific variations in antiemetic effectiveness that reflect pharmacokinetic and administration-route differences rather than population heterogeneity. Conclusions: This real-world analysis demonstrates that antiemetic effectiveness varies by CINV phase, even within a relatively homogeneous, high-risk patient cohort. The results highlight the importance of phase-tailored prophylactic strategies to optimize symptom control and improve patient quality of life in highly emetogenic chemotherapy settings.

## Simple Summary

Nausea and vomiting caused by chemotherapy are among the most troublesome side effects for cancer patients and can significantly reduce quality of life and willingness to continue treatment. Several medications are used to prevent these symptoms, but their effectiveness may vary depending on the timing after chemotherapy. In this study, we compared two commonly used anti-nausea drugs in women with breast cancer receiving intensive chemotherapy. We found that one drug was more effective in preventing nausea and vomiting shortly after chemotherapy, while the other provided better protection several days later. These findings suggest that choosing anti-nausea treatment according to the timing of symptoms may improve patient comfort and treatment experience in routine clinical practice.

## 1. Introduction

Chemotherapy-induced nausea and vomiting (CINV) is one of the most distressing adverse effects of cancer treatment, substantially impairing patients’ quality of life and potentially jeopardizing curative outcomes by reducing treatment adherence [1,2,3]. Despite notable progress in antiemetic therapy, CINV remains a major clinical challenge in patients receiving highly emetogenic chemotherapy (HEC), occurring in approximately 60–80% of cases [3,4]. The development of CINV depends not only on the emetogenic potential of the chemotherapy regimen but also on individual patient-related factors, including age, sex, history of alcohol use, susceptibility to motion sickness, and previous experiences of nausea [1,5,6].

International antiemetic guidelines define CINV as a biphasic clinical condition with temporally and clinically distinct symptom patterns. The Multinational Association of Supportive Care in Cancer/European Society for Medical Oncology (MASCC/ESMO) and the American Society of Clinical Oncology (ASCO) guideline updates specify that the acute phase, occurring within the first 24 h (0–24 h) after chemotherapy administration, is marked by early onset of nausea and vomiting that typically peaks on the day of treatment. In contrast, the delayed phase develops beyond the first day, generally spanning 24–120 h, and is characterized by a more protracted and less predictable clinical course [7,8]. Clinical evidence further indicates that delayed-phase symptoms remain more difficult to control in routine practice, even with adherence to guideline-based prophylactic strategies, highlighting the importance of distinguishing between these phases in both clinical assessment and treatment planning [9].

Biologically, the acute phase is primarily mediated by serotonin release from enterochromaffin cells in the gastrointestinal tract, leading to activation of 5-hydroxytryptamine type-3 (5-HT_3_) receptors. In contrast, the delayed phase is mainly associated with sustained substance P-mediated neurokinin-1 (NK-1) receptor activation within the central nervous system [10]. These mechanistic differences provide a biological rationale for phase-adapted antiemetic strategies and help to explain the ongoing challenges in achieving optimal control of delayed-phase CINV [3,10].

Sex and age are among the most influential patient-related risk factors for the development of CINV. A substantial body of evidence indicates that the incidence and severity of CINV are significantly higher in women and in younger patients, particularly those under 50 years of age, compared with men and older individuals [1,5,6,7,8]. These differences are thought to reflect a complex interaction among biological, hormonal, and neurochemical factors, including variations in neurotransmitter sensitivity and activation of emetogenic pathways [10]. Consequently, female sex and younger age are consistently recognized as key determinants of CINV risk and are widely regarded as markers of a high-risk population requiring careful antiemetic prophylaxis [1,5,6,7,8,10].

The involvement of distinct pathophysiological mechanisms in the development of CINV during the acute and delayed phases contributes to differences in antiemetic control rates [10]. In particular, the predominance of serotonergic pathways in the acute phase, and substance P-mediated NK-1 receptor activation in the delayed phase, result in variable responsiveness to standard antiemetic regimens. Consequently, these mechanistic differences underscore the need for phase-specific antiemetic strategies to achieve effective prevention of nausea and vomiting across both phases of chemotherapy treatment [10].

The majority of breast cancer cases occur in women, and in Türkiye, breast cancer is diagnosed at a relatively younger age, with the median age at diagnosis reported to be below 50 years, in contrast to many Western populations where the median age is typically higher [11]. Anthracycline–cyclophosphamide (AC)-based chemotherapy, classified as a highly emetogenic regimen, remains one of the standard treatment approaches widely used in patients with non-metastatic breast cancer as part of curative-intent treatment strategies in the adjuvant and neoadjuvant settings [12]. Consequently, patients receiving AC-based HEC represent a clinically homogeneous population characterized by multiple established risk factors for chemotherapy-induced nausea and vomiting.

This population provides an appropriate clinical model for the accurate evaluation of differences in antiemetic responses, particularly across the acute and delayed phases of CINV. Despite advances in antiemetic prophylaxis, maintaining treatment adherence and preserving quality of life remain significant challenges in this high-risk group [2,3]. Studies conducted in such homogeneous patient cohorts may help to clarify the phase-specific dynamics of CINV and provide a scientific basis for optimizing antiemetic strategies. Nevertheless, real-world evidence evaluating antiemetic efficacy across different CINV phases in this high-risk population remains limited.

Accordingly, this retrospective real-world study aimed to systematically evaluate phase-specific differences in antiemetic efficacy between the acute and delayed phases in women with breast cancer receiving AC-based HEC administered with curative intent. In addition, the study sought to assess the real-world performance of guideline-based antiemetic regimens in this clinically homogeneous, high-risk population and to identify potential gaps in symptom control across different phases of CINV. By focusing on a well-defined patient cohort treated with standardized chemotherapy protocols, this study aims to contribute clinically relevant evidence to support the optimization of phase-adapted, practically applicable antiemetic strategies in routine oncology practice.

## 2. Materials and Methods

### 2.1. Study Design and Patient Selection

This study was designed as a single-center, retrospective, observational analysis conducted in a real-world clinical setting. The study population consisted of women diagnosed with stage II–III breast cancer who were treated between January 2023 and December 2025 and received an AC-based HEC regimen as part of adjuvant or neoadjuvant treatment with curative intent. The AC regimen consisted of doxorubicin at a dose of 60 mg/m^2^ and cyclophosphamide at a dose of 600 mg/m^2^, administered according to institutional standard protocols. This regimen is widely recommended by international guidelines as a standard chemotherapy approach for patients with non-metastatic breast cancer [12].

Eligible patients were identified through institutional chemotherapy records and electronic medical databases. Only patients who received the first cycle of the AC-based HEC regimen were included in order to ensure a homogeneous assessment of CINV without potential confounding effects from prior chemotherapy exposure or cumulative toxicity. Patient-reported outcomes related to CINV were assessed using the MASCC antiemetic questionnaire, which was completed during routine outpatient follow-up.

Inclusion Criteria

Patients were eligible for inclusion if they met all of the following criteria:

Age ≥ 18 years at the time of chemotherapy initiation;

Histopathologically confirmed stage II–III breast cancer;

Receipt of the first cycle of an AC-based HEC regimen in the adjuvant or neoadjuvant setting;

Complete and correctly filled MASCC antiemetic questionnaire, with no missing data.

Exclusion Criteria

Patients were excluded from the analysis if they met any of the following criteria:

Male sex;

Diagnosis of a malignancy other than breast cancer;

Prior exposure to systemic anticancer therapy, including chemotherapy or antiemetic prophylaxis;

Inability to complete the questionnaire due to illiteracy or cognitive impairment;

Incomplete or incorrectly completed MASCC questionnaire.

### 2.2. Data Collection and Assessment

Patient data were retrospectively collected from institutional electronic medical records and Multinational Association of Supportive Care in Cancer (MASCC) Anti-emesis Tool (MAT) questionnaires. The MAT is a validated, reliable, patient-reported outcome instrument specifically developed to assess both the frequency and severity of CINV in routine clinical practice [13]. It allows for standardized evaluation of nausea and vomiting across different phases of chemotherapy and has been widely used in both clinical studies and real-world settings. At our institution, the MAT questionnaire is routinely administered as part of standard supportive care to patients receiving HEC, independent of study participation. For the purposes of this analysis, only patients who completed the MAT during their first cycle of AC-based HEC were included, thereby minimizing potential confounding effects related to prior chemotherapy exposure, anticipatory nausea, or cumulative toxicity. The questionnaire was administered at the first scheduled outpatient follow-up visit, which typically occurred approximately one week after chemotherapy administration, enabling assessment of both acute and delayed CINV symptoms. This timing reflects real-world clinical practice, where symptom evaluation is commonly performed during early post-treatment follow-up visits rather than through daily prospective monitoring.

The MAT questionnaire was administered to patients on the day of chemotherapy administration and subsequently collected approximately one week later during the routine outpatient follow-up visit. This approach allowed patients to report symptoms experienced throughout the entire risk period for chemotherapy-induced nausea and vomiting, consistent with real-world clinical practice. The MAT separately evaluates the frequency and severity of nausea and vomiting during the acute phase (0–24 h) and the delayed phase (24–120 h) following chemotherapy, enabling a phase-specific assessment of symptom burden. In addition to patient-reported outcomes, demographic and clinical variables were retrospectively extracted from institutional medical records. These variables included age, menopausal status, pregnancy history, and established patient-related risk factors for CINV, such as a history of hyperemesis gravidarum, susceptibility to motion sickness, and the presence of comorbid medical conditions. Collection of these variables was performed to allow a comprehensive characterization of the study population and to facilitate exploratory analyses of factors potentially associated with variability in antiemetic response across different CINV phases.

Adverse events were retrospectively assessed through a comprehensive review of electronic medical records, including outpatient clinic notes, hospitalization records, and treatment modification reports. This approach allowed for the identification of adverse events occurring during routine clinical care without protocol-driven reporting requirements. Clinically significant adverse events were defined as those that required hospitalization, led to treatment discontinuation, necessitated dose modification, or prompted additional medical intervention. Only events deemed clinically relevant and clearly documented in the medical records were included in the analysis, in order to ensure consistency and reliability of adverse event assessment in this real-world setting.

### 2.3. Study Endpoints

To evaluate the control of CINV, three pre-defined clinical endpoints were analyzed in this study. These endpoints were selected to reflect both the effectiveness of prophylactic antiemetic therapy and the need for additional symptom management in routine clinical practice.

The primary and secondary endpoints were defined as follows: complete response (CR), defined as the absence of any vomiting episodes and no requirement for additional (rescue) antiemetic medication during the assessment period; no vomiting, defined as the complete absence of vomiting episodes regardless of nausea severity; and use of rescue therapy, defined as the requirement for additional antiemetic treatment beyond the planned prophylactic regimen. Each endpoint was evaluated separately and independently across three predefined post-chemotherapy time intervals to allow a phase-specific assessment of antiemetic efficacy: the acute phase (0–24 h), corresponding to the first 24 h following chemotherapy administration; the delayed phase (24–120 h), defined as the period extending from 24 h up to five days after chemotherapy; and the overall phase (0–120 h), representing the combined acute and delayed assessment period.

These phase definitions and endpoint categorizations were standardized in accordance with international antiemetic guidelines, including those of the MASCC/ESMO and the NCCN, ensuring consistency with established clinical and research frameworks [1,9].

### 2.4. Treatment Regimens

All patients received triple antiemetic prophylaxis prior to initiation of the HEC protocol, in accordance with the standard supportive care practices of our institution. Antiemetic prophylaxis regimens were implemented based on the recommendations of the NCCN [1] and MASCC/ESMO guidelines [7]. Each regimen consisted of a 5-HT_3_ receptor antagonist, dexamethasone, and a NK-1 receptor antagonist, forming the guideline-recommended standard of care for patients receiving HEC.

Patients were treated with one of two NK-1 receptor antagonist-based antiemetic protocols routinely used in daily clinical practice at our center. The choice of regimen was determined by routine clinical decision-making rather than by study allocation.

Patients in the fosaprepitant group received a single intravenous dose of fosaprepitant (150 mg) on the day of chemotherapy in combination with a 5-HT3 receptor antagonist (ondansetron or palonosetron) and dexamethasone according to institutional standards. Patients in the aprepitant group received oral aprepitant over three consecutive days (125 mg on day 1, followed by 80 mg on days 2 and 3) in combination with a 5-HT3 receptor antagonist and dexamethasone, consistent with guideline-based recommendations.

Both antiemetic regimens were administered as standard triple prophylactic protocols recommended by international guidelines for the prevention of CINV associated with HEC. The specific doses, schedules, and combinations of the antiemetic agents used in this study are summarized in Table 1.

### 2.5. Statistical Analysis

All statistical analyses were performed using SPSS Statistics for Windows, Version 28.0 (IBM Corp., Armonk, NY, USA). Continuous variables were summarized using descriptive statistics, including mean ± standard deviation for normally distributed data and median (minimum–maximum) for non-normally distributed data. Categorical variables were presented as absolute numbers and percentages (%).

The distribution of continuous variables was assessed using both the Kolmogorov–Smirnov and Shapiro–Wilk tests in order to determine the appropriateness of parametric or non-parametric statistical methods. Based on the results of normality testing, non-parametric tests were applied for between-group comparisons.

For comparisons between study groups, the Mann–Whitney U test was used to compare independent numerical variables, and the chi-square (X^2^) test was applied to evaluate associations between categorical variables.

All statistical tests were two-sided, and a *p*-value < 0.05 was considered to indicate statistical significance.

### 2.6. Ethical Approval

This study was approved by the Ethics Committee of the University of Health Sciences Sancaktepe Şehit Prof. Dr. İlhan Varank Training and Research Hospital (decision no. 2024/253, approval date: 15 May 2024). Owing to the retrospective design of the study, the requirement for informed consent was waived by the Ethics Committee. All data were collected and analyzed in accordance with the principles of confidentiality and in compliance with the Personal Data Protection Law of Türkiye (Law No. 6698).

### 2.7. Use of Language Assistance

During manuscript preparation, a generative artificial intelligence (GenAI) tool (ChatGPT, OpenAI; GPT-5.2 version) was used solely for language editing and stylistic refinement. All scientific content, including the study design, data analysis, interpretation of results, and final conclusions, was entirely developed, verified, and approved by the authors. The authors take full responsibility for the integrity and accuracy of the manuscript.

## 3. Results

### 3.1. Patients

A total of 260 female patients were included in the study. Of these, 133 patients (51.2%) received an aprepitant-based antiemetic regimen, while 127 patients (48.8%) received a fosaprepitant-based regimen. The median age of the overall cohort was 46 years (range: 32–74 years), and 38.5% of patients were postmenopausal at the time of treatment initiation.

Regarding disease characteristics, the majority of patients were diagnosed with stage II breast cancer (64.6%), while 35.4% had stage III disease. The predominant histopathological subtype was invasive ductal carcinoma, accounting for 87.7% of cases. In terms of treatment setting, 84.6% of patients received chemotherapy in the neoadjuvant setting, whereas 15.4% were treated in the adjuvant setting. Additionally, a dose-dense AC regimen was administered to 193 patients (74.2%), reflecting routine clinical practice for high-risk, non-metastatic breast cancer.

Comparative analysis of baseline characteristics revealed that the postmenopausal rate was significantly higher in the fosaprepitant group (*p* < 0.05). In contrast, the frequency of invasive lobular carcinoma was significantly higher in the aprepitant group (*p* < 0.05). No statistically significant differences were observed between the two treatment groups with respect to other demographic or clinicopathological variables (*p* > 0.05), indicating a broadly comparable baseline profile between groups (Table 2 and Table 3).

In addition, the rates of motion sickness and pregnancy history were significantly higher in the aprepitant group (*p* < 0.05), whereas the incidence of hyperemesis gravidarum did not differ significantly between the two groups (*p* > 0.05). The requirement for rescue antiemetic therapy was significantly higher in the fosaprepitant group (*p* < 0.05).

With respect to molecular subtypes, the HR+/HER2− subtype was significantly more frequent in the fosaprepitant group, while the HR+/HER2+ subtype was more common in the aprepitant group (*p* < 0.05).

### 3.2. Efficacy

#### 3.2.1. Complete Response (CR)

**Figure 1:**
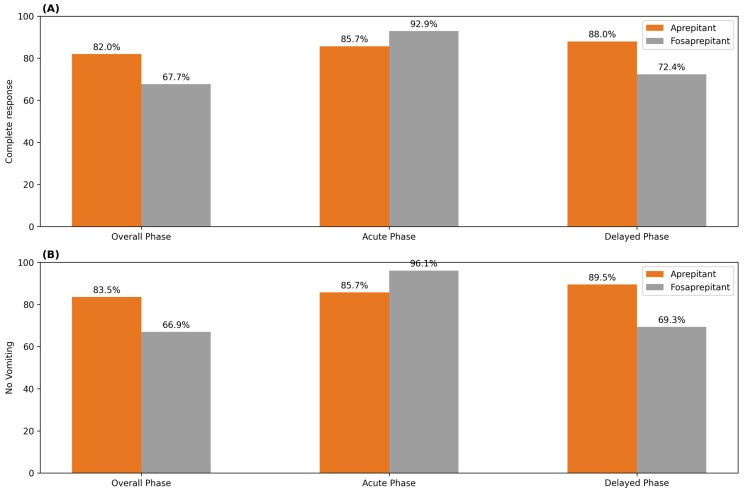
Complete response (CR) and no vomiting (NV). The Bar graph shows the percentage of patients achieving (**A**) CR and (**B**) NV endpoints during the 120 h after chemotherapy initiation. Gray bars represent a single-dose fosaprepitant regimen; orange bars, a 3-day aprepitant regimen in this study. OP was 0 to 120 h after initiation of chemotherapy. AP was 0 to 24 h after initiation of chemotherapy. DP was 24 to 120 h after initiation of chemotherapy.

In the overall phase (0–120 h), the CR rate was 67.7% in the fosaprepitant group and 82.0% in the aprepitant group, with a statistically significant difference favoring aprepitant (*p* = 0.008). During the acute phase (0–24 h), CR was achieved in 92.9% of patients receiving fosaprepitant and 85.7% of those receiving aprepitant; however, this difference did not reach statistical significance (*p* = 0.061). In contrast, during the delayed phase (24–120 h), the CR rate was significantly higher in the aprepitant group compared with the fosaprepitant group (88.0% vs. 72.4%, *p* = 0.002). Overall, the aprepitant-based regimen was associated with significantly higher CR rates in the delayed and overall phases, whereas the fosaprepitant-based regimen demonstrated a numerical advantage in the acute phase, although this difference was not statistically significant (Figure 1, Table 4).

#### 3.2.2. No Vomiting

In the overall phase, the proportion of patients with no vomiting was 66.9% in the fosaprepitant group and 83.5% in the aprepitant group, demonstrating a statistically significant difference in favor of aprepitant (*p* = 0.002). During the acute phase, the absence of vomiting was observed more frequently in the fosaprepitant group compared with the aprepitant group (96.1% vs. 85.0%, *p* = 0.004). Conversely, in the delayed phase, the aprepitant-based regimen was associated with a significantly higher rate of no vomiting than the fosaprepitant-based regimen (89.5% vs. 69.3%, *p* < 0.001). Overall, these findings indicate that fosaprepitant provides superior control of vomiting during the acute phase, whereas aprepitant demonstrates greater efficacy in preventing vomiting during the delayed and overall phases (Figure 1, Table 4).

### 3.3. Rescue Therapy and Safety

The use of rescue antiemetic therapy was significantly higher in the fosaprepitant group (*p* < 0.05). While the fosaprepitant-based regimen was associated with better control during the acute phase, its effectiveness decreased during the delayed phase, during which the aprepitant-based regimen demonstrated superior control of emesis.

Both antiemetic regimens were well tolerated. No clinically significant adverse events requiring hospitalization, treatment discontinuation, or dose modification were observed in either treatment group.

## 4. Discussion

This study provides a comparative real-world evaluation of the effectiveness of fosaprepitant- and aprepitant-based antiemetic regimens in patients with breast cancer receiving HEC. The rates of CR and absence of vomiting observed in our cohort were largely consistent with previously published data [14,15,16,17,18], supporting both the robustness of our findings and the reliability of real-world evidence in this setting. Our results indicate that aprepitant shows greater efficacy during the delayed and overall phases. In contrast, fosaprepitant tends to achieve higher CR rates in the acute phase and appears more effective at preventing vomiting. These phase-specific differences underscore the need for individualized antiemetic strategies rather than a uniform approach for all patients. Nevertheless, the study’s retrospective design and imbalances in certain baseline risk factors (such as a history of motion sickness or hyperemesis gravidarum) warrant cautious interpretation of the results.

In our study, fosaprepitant demonstrated higher control rates during the acute phase. The CR rate was 92.9% in the fosaprepitant group compared with 85.7% in the aprepitant group, and the rate of no vomiting was significantly higher in the fosaprepitant group (96.1% vs. 85.0%). These findings indicate that fosaprepitant provides stronger protection against nausea and vomiting during the first 24 h after chemotherapy and are consistent with the existing literature [15,16,18]. However, some randomized controlled trials have reported that fosaprepitant does not confer additional benefit in the acute phase [17]. This discrepancy may stem from the controlled nature of randomized trials, in contrast with real-world practice, where numerous confounding factors influence outcomes. The results observed in our study may be attributed to the intravenous administration of fosaprepitant, which enables rapid receptor blockade and high early bioavailability [19]. Given these characteristics, fosaprepitant may be a suitable option for patients who require prompt symptom control during the acute phase.

Aprepitant demonstrated higher efficacy than fosaprepitant during the delayed and overall phases in our study. The CR rate in the delayed phase was 88.0% in the aprepitant group versus 72.4% in the fosaprepitant group, and a similar significant difference favoring aprepitant was observed in the overall phase (82.0% vs. 67.7%). These findings suggest that aprepitant is more effective in controlling nausea and vomiting that emerge particularly after the second day following chemotherapy. Some studies have suggested a potential advantage of aprepitant-based regimens in the delayed phase, although most randomized trials report comparable efficacy between aprepitant and fosaprepitant; in our real-world cohort, aprepitant was associated with higher delayed- and overall-phase CR rates [4,5,6,20]. The advantage of aprepitant in this phase may be explained by the sustained NK-1 receptor blockade achieved with additional doses on days two and three. Prior studies have shown that administering an extra dose of fosaprepitant on day three provides better control of delayed-phase nausea and vomiting than a single-dose regimen [21,22]. This supports the hypothesis that extended NK-1 receptor blockade contributes to sustained antiemetic efficacy. Overall, the findings indicate that aprepitant may offer a clinical advantage for patients at high risk of delayed-phase nausea and vomiting.

Although our study was conducted in a relatively homogeneous patient population in terms of sex, disease stage, cancer type, and chemotherapy regimen, the observed individual variations in nausea and vomiting frequency and in response to antiemetic therapy underscore the importance of personalized approaches to CINV prophylaxis. Taken together, these findings suggest that the differing pharmacokinetic properties of fosaprepitant and aprepitant translate into distinct clinical advantages, with each agent offering phase-specific benefits. Our real-world data highlight these phase-specific differences in efficacy and reinforce the need for individualized decision-making when selecting antiemetic therapy.

## 5. Limitations and Future Directions

This study has several limitations that should be acknowledged. The retrospective design and single-center setting represent the primary limitations and may restrict the generalizability of the findings. In addition, because the MAT was completed several days after chemotherapy administration, the possibility of recall bias, particularly with respect to delayed-phase nausea, cannot be fully excluded.

Although the study population was intentionally designed to be relatively homogeneous by focusing on a single sex, cancer type, and chemotherapy regimen, some baseline imbalances in patient-related risk factors were observed between treatment groups. Differences in menopausal status, histologic subtype, and susceptibility to motion sickness, all of which have been reported to influence the risk of CINV, may have contributed to variability in antiemetic outcomes and should be considered when interpreting the results.

Furthermore, the use of other NK-1 receptor antagonists, olanzapine, and additional antiemetic agents is not routine in clinical practice in our country, which limited the ability to perform comparisons with these agents.

Despite these limitations, the study has several strengths, including the evaluation of a clinically well-defined, high-risk population treated with standardized chemotherapy protocols, allowing for a focused assessment of phase-specific antiemetic efficacy in a real-world setting.

Future multicenter, prospective studies incorporating broader antiemetic options and more detailed adjustment for patient-related risk factors may help to further refine personalized and phase-adapted antiemetic strategies and support the development of updated clinical guidelines.

## 6. Conclusions

In this real-world study of women with breast cancer receiving anthracycline–cyclophosphamide (AC)-based highly emetogenic chemotherapy, both fosaprepitant- and aprepitant-based triple antiemetic regimens were effective in preventing chemotherapy-induced nausea and vomiting. Importantly, their antiemetic efficacy differed according to the phase of CINV. Fosaprepitant provided better protection during the acute phase, particularly with respect to early control of symptoms, whereas aprepitant demonstrated superior efficacy during the delayed phase.

These findings highlight the importance of considering both the acute and delayed phases of CINV when selecting antiemetic prophylaxis in routine clinical practice. A phase-adapted approach, rather than a uniform strategy for all patients, may allow for more effective symptom control. Tailoring antiemetic regimens to the dominant mechanisms of each phase has the potential to improve patient comfort, reduce the need for rescue therapy, and support adherence to potentially curative chemotherapy, ultimately leading to better overall clinical outcomes.

## Figures and Tables

**Table 1:** Standard Antiemetic Regimens.

Fosaprepitant-Based Regimen		
Day 1	Day 2	Day 3
Fosaprepitant 150 mg IV Ondansetron 8 mg IV Dexamethasone 12 mg IV	Dexamethasone 8 mg PO	Dexamethasone 8 mg PO
Aprepitant-Based Regimen		
Day 1	Day 2	Day 3
Aprepitant 125 mg PO Ondansetron 8 mg IV Dexamethasone 12 mg IV	Aprepitant 80 mg PO Dexamethasone 8 mg PO	Aprepitant 80 mg PO Dexamethasone 8 mg PO

**Table 2:** Baseline Demographics and Clinical Characteristics.

Variable	n	%
Age (years, median min-max)	46 (32–74)	
Menopausal Status		
Menopausal	100	38.5
PreMenopausal	136	52.3
PeriMenopausal	24	9.2
Comorbidities		
Absent	200	76.9
Present	60	23.1
ECOG PS		
0	240	92.3
1	20	7.7
Histological Type		
IDC	228	87.7
ILC	16	6.2
Mixed	8	3.1
Others	8	3.1
Histological Subtype		
HR + HER2−	124	47.7
HR + HER2+	72	27.7
HR − HER2−	36	13.8
HR − HER2+	28	10.8
Breast Laterality		
Right	120	46.2
Left	132	50.8
Bilateral	8	3.1
Stage		
II	168	64.6
III	92	35.4
Adjuvant/Neoadjuvant		
Adjuvant	40	15.4
Neoadjuvant	220	84.6
Chemotherapy Regimen		
AC	67	25.8
ddAC	193	74.2
Motion Sickness		
Absent	153	58.8
Present	107	41.2
Pregnancy History		
No	32	12.3
Yes	228	87.7
History of HG		
No	69	30.8
Yes	155	69.2
Unknown	4	-
Antiemetic Agent Used		
Aprepitant	133	51.2
Fosaprepitant	127	48.8
Analgesic Use		
No	108	41.5
Yes	114	43.8
Unknown	38	14.6
Type of Analgesic Used		
NSAIDs	10	8.8
Paracetamol	6	5.3
Narcotic Analgesics	4	3.5
Unknown	94	82.5
G-CSF Use		
No	62	23.8
Yes	198	76.2
Use of Rescue Therapy		
No	241	92.7
Yes	19	7.3

IDC: Invasive Ductal Carcinoma; ILC: Invasive Lobular Carcinoma; HR: Hormone Receptor; HER2: Human Epidermal Growth Factor Receptor 2; ECOG PS: Eastern Cooperative Oncology Group Performance Status; AC: Doxorubicin and Cyclophosphamide; ddAC: Dose-Dense AC; HG: Hyperemesis Gravidarum; NSAIDs: Non-Steroidal Anti-Inflammatory Drugs; G-CSF: Granulocyte Colony-Stimulating Factor.

**Table 3:** Comparison of Baseline Characteristics Between Aprepitant and Fosaprepitant Groups.

Variable	Aprepitant Group (n = 133)	Fosaprepitant Group (n = 127)	*p*-Value
Age (years, mean ± SD)	48.3 ± 10.9	48.0 ± 10.4	0.971 (m)
Menopausal Status			0.007 (X^2^)
Menopausal	40 (30.1%)	60 (47.2%)	
Pre-Menopausal	76 (57.1%)	60 (47.2%)	
Peri-Menopausal	17 (12.8%)	7 (5.5%)	
Comorbidities			0.428 (X^2^)
Absent	105 (78.9%)	95 (74.8%)	
Present	28 (21.1%)	32 (25.2%)	
ECOG PS			0.410 (X^2^)
0	121 (91.0%)	119 (93.7%)	
1	12 (9.0%)	8 (6.3%)	
Histological Type			
IDC	113 (85.0%)	115 (90.6%)	0.170 (X^2^)
ILC	16 (12.0%)	0 (0.0%)	0.000 (X^2^)
ILC IDC Mixed	0 (0.0%)	8 (6.3%)	0.003 (X^2^)
Others	4 (3.0%)	4 (3.1%)	0.947 (X^2^)
Histological Subtype			
HR + HER2−	50 (37.6%)	74 (58.3%)	0.001 (X^2^)
HR + HER2+	48 (36.1%)	24 (18.9%)	0.002 (X^2^)
HR − HER2−	16 (12.0%)	20 (15.7%)	0.386 (X^2^)
HR − HER2+	19 (14.3%)	9 (7.1%)	0.061 (X^2^)
Breast Laterality			0.252 (X^2^)
Right	68 (51.1%)	52 (40.9%)	
Left	61 (45.9%)	71 (55.9%)	
Bilateral	4 (3.0%)	4 (3.1%)	
Stage			0.123 (X^2^)
II	80 (60.2%)	88 (69.3%)	
III	53 (39.8%)	39 (30.7%)	
Adjuvant/Neoadjuvant Therapy			0.397 (X^2^)
Adjuvant	18 (13.5%)	22 (17.3%)	
Neoadjuvant	115 (86.5%)	105 (82.7%)	
Chemotherapy Regimen			0.519 (X^2^)
AC	32 (24.1%)	35 (27.6%)	
ddAC	101 (75.9%)	92 (72.4%)	
Motion Sickness			<0.001 (X^2^)
Absent	64 (48.1%)	89 (70.1%)	
Present	69 (51.9%)	38 (29.9%)	
Pregnancy History			0.002 (X^2^)
No	8 (6.0%)	24 (18.9%)	
Yes	125 (94.0%)	103 (81.1%)	
History of HG			0.465 (X^2^)
No	36 (28.8%)	33 (33.3%)	
Yes	89 (71.2%)	66 (66.7%)	
Analgesic Use			0.195 (X^2^)
No	68 (52.3%)	40 (43.5%)	
Yes	62 (47.7%)	52 (56.5%)	
G-CSF Use			0.279 (X^2^)
No	28 (21.1%)	34 (26.8%)	
Yes	105 (78.9%)	93 (73.2%)	
Use of Rescue Therapy			0.024 (X^2^)
No	128 (96.2%)	113 (89.0%)	
Yes	5 (3.8%)	14 (11.0%)	

m: Mann–Whitney U test; X^2^: Chi-square test; IDC: Invasive Ductal Carcinoma; ILC: Invasive Lobular Carcinoma; HR: Hormone Receptor; HER2: Human Epidermal Growth Factor Receptor 2; ECOG PS: Eastern Cooperative Oncology Group Performance Status; AC: Doxorubicin and Cyclophosphamide; ddAC: Dose-Dense AC.

**Table 4:** Comparison of Vomiting and Use of Rescue Therapy Between Aprepitant and Fosaprepitant Groups.

Variable	Aprepitant Group	Fosaprepitant Group	
	n = 133 (%)	n = 127 (%)	*p*-Value
CR			
Overall Phase	109 (82.0)	86 (67.7)	0.008 (X^2^)
Acute Phase	114 (85.7)	118 (92.9)	0.061 (X^2^)
Delayed Phase	117 (88.0)	92 (72.4)	0.002 (X^2^)
No Vomiting			
Overall Phase	111 (83.5)	85 (66.9)	0.002 (X^2^)
Acute Phase	114 (85.7)	122 (96.1)	0.004 (X^2^)
Delayed Phase	119 (89.5)	88 (69.3)	<0.001(X^2^)

X^2^: chi-square test. CR: complete response (no vomiting and no rescue antiemetic medication); NV: no vomiting.

## Data Availability

The data supporting the reported results are available in the corresponding author’s database. Due to the study’s retrospective nature and privacy considerations, the data are not publicly available. Requests for access to the data can be made to the corresponding author upon reasonable request.

## Abbreviations

The following abbreviations are used in this manuscript:

CINV	Chemotherapy-induced nausea and vomiting
HEC	Highly emetogenic chemotherapy
5-HT_3_	5-hydroxytryptamine type 3
NK-1	Neurokinin-1 (NK-1)
MASCC	Multinational Association of Supportive Care in Cancer
MAT	MASCC Antiemesis Tool
m	Mann–Whitney U test
X^2^	Chi-square test
IDC	Invasive Ductal Carcinoma
ILC	Invasive Lobular Carcinoma
HR	Hormone Receptor
HER2	Human Epidermal Growth Factor Receptor 2
ECOG PS	Eastern Cooperative Oncology Group Performance Status
AC	Doxorubicin and Cyclophosphamide
ddAC	Dose-Dense Doxorubicin and Cyclophosphamide
G-CSF	Granulocyte Colony-Stimulating Factor
CR	Complete Response
NV	No Vomiting
SPSS	Statistical Package for the Social Sciences

## References

[1] National Comprehensive Cancer Network (NCCN) (2025). NCCN Clinical Practice Guidelines in Oncology (NCCN Guidelines^®^): Antiemesis. Version 2.2025. National Comprehensive Cancer Network. https://www.nccn.org.

[2] Razvi Y., Chan S., McFarlane T., McKenzie E., Zaki P., DeAngelis C., Pidduck W., Bushehri A., Chow E., Jerzak K.J. (2019). Asco, nccn, mascc/esmo: A comparison of antiemetic guidelines for the treatment of chemotherapy-induced nausea and vomiting in adult patients. Support. Care Cancer.

[3] Sommariva S., Pongiglione B., Tarricone R. (2016). Impact of chemotherapy-induced nausea and vomiting on health-related quality of life and resource utilization: A systematic review. Crit. Rev. Oncol. Hematol..

[4] Aapro M., Carides A., Rapoport B.L., Schmoll H.J., Zhang L., Warr D. (2015). Aprepitant and fosaprepitant: A 10-year review of efficacy and safety. Oncologist.

[5] Campos D., Pereira J.R., Reinhardt R.R., Carracedo C., Poli S., Vogel C., Martinez-Cedillo J., Erazo A., Wittreich J., Eriksson L.O. (2001). Prevention of cisplatin-induced emesis by the oral neurokinin-1 antagonist, mk-869, in combination with granisetron and dexamethasone or with dexamethasone alone. J. Clin. Oncol..

[6] Di Maio M., Baratelli C., Bironzo P., Vignani F., Bria E., Sperti E., Marcato M., Roila F. (2018). Efficacy of neurokinin-1 receptor antagonists in the prevention of chemotherapy-induced nausea and vomiting in patients receiving carboplatin-based chemotherapy: A systematic review and meta-analysis. Crit. Rev. Oncol. Hematol..

[7] Herrstedt J., Clark-Snow R., Ruhlmann C.H., Molassiotis A., Olver I., Rapoport B.L., Aapro M., Dennis K., Hesketh P.J., Navari R.M. (2024). 2023 mascc and esmo guideline update for the prevention of chemotherapy- and radiotherapy-induced nausea and vomiting. ESMO Open.

[8] Hesketh P.J., Kris M.G., Basch E., Bohlke K., Barbour S.Y., Clark-Snow R.A., Danso M.A., Dennis K., Dupuis L.L., Dusetzina S.B. (2020). Antiemetics: Asco guideline update. J. Clin. Oncol..

[9] Becherini C., Salvestrini V., Desideri I., Vagnoni G., Bonaparte I., Bertini N., Mattioli C., Angelini L., Visani L., Scotti V. (2024). Impact of fosaprepitant in the prevention of nausea and emesis in head and neck cancer patients undergoing cisplatin-based chemoradiation: A pilot prospective study and a review of literature. Radiol. Med..

[10] Navari R.M., Aapro M. (2016). Antiemetic prophylaxis for chemotherapy-induced nausea and vomiting. N. Engl. J. Med..

[11] Bray F., Laversanne M., Sung H., Ferlay J., Siegel R.L., Soerjomataram I., Jemal A. (2024). Global cancer statistics 2022: Globocan estimates of incidence and mortality worldwide for 36 cancers in 185 countries. CA Cancer J. Clin..

[12] National Comprehensive Cancer Network (2025). NCCN Clinical Practice Guidelines in Oncology: Breast Cancer. Version 5.2025, National Comprehensive Cancer Network. https://www.nccn.org/guidelines/category_1.

[13] Multinational Association of Supportive Care in Cancer: Mascc Antiemesis Tool Turkish. https://mascc.org/wp-content/uploads/2022/04/mat_turkish_tool.pdf.

[14] Ando Y., Hayashi T., Ito K., Suzuki E., Mine N., Miyamoto A., Oya M., Matsuda H., Isaji A., Nakanishi T. (2016). Comparison between 5-day aprepitant and single-dose fosaprepitant meglumine for preventing nausea and vomiting induced by cisplatin-based chemotherapy. Support. Care Cancer.

[15] Grunberg S., Chua D., Maru A., Dinis J., DeVandry S., Boice J.A., Hardwick J.S., Beckford E., Taylor A., Carides A. (2011). Single-dose fosaprepitant for the prevention of chemotherapy-induced nausea and vomiting associated with cisplatin therapy: Randomized, double-blind study protocol-ease. J. Clin. Oncol..

[16] He L., Wang J., Pu W., Li H., Liu B., Wang Z., Han Q., Wang Y., Xu B., Hu J. (2025). Prospective clinical evidence from over 1,000 pan-cancer patients: A complement to fosaprepitant in the prevention of chemotherapy-induced nausea and vomiting. BMC Cancer.

[17] Weinstein C., Jordan K., Green S.A., Camacho E., Khanani S., Beckford-Brathwaite E., Vallejos W., Liang L.W., Noga S.J., Rapoport B.L. (2016). Single-dose fosaprepitant for the prevention of chemotherapy-induced nausea and vomiting associated with moderately emetogenic chemotherapy: Results of a randomized, double-blind phase iii trial. Ann. Oncol..

[18] Yang L.Q., Sun X.C., Qin S.K., Cheng Y., Shi J.H., Chen Z.D., Wang Q.M., Zhang H.L., Hu B., Liu B. (2017). Efficacy and safety of fosaprepitant in the prevention of nausea and vomiting following highly emetogenic chemotherapy in chinese people: A randomized, double-blind, phase iii study. Eur. J. Cancer Care.

[19] Agency E.M. (2025). Fosaprepitant Smpc (Emend IV). European Medicines Agency. https://www.ema.europa.eu/en/medicines.

[20] Zhang Z., Yang Y., Lu P., Li X., Chang J., Zheng R., Zhou L., Chen S., Chen X., Ren B. (2020). Fosaprepitant versus aprepitant in the prevention of chemotherapy-induced nausea and vomiting in patients receiving cisplatin-based chemotherapy: A multicenter, randomized, double-blind, double-simulated, positive-controlled phase iii trial. Ann. Transl. Med..

[21] Gao A., Guan S., Sun Y., Wang L., Meng F., Liu X., Gu L., Li G., Zhong D., Zhang L. (2023). Prolonged usage of fosaprepitant for prevention of delayed chemotherapy-induced nausea and vomiting(CINV) in patients receiving highly emetogenic chemotherapy. BMC Cancer.

[22] Li Y., Wan Y., Yang X., Chen P., Gui Y., He L., Xie Y., Tian J., Duan P., Liu G. (2024). Two doses of fosaprepitant included prophylactic treatment for the three-day cisplatin-based chemotherapy induced nausea and vomiting. J. Cancer Res. Clin. Oncol..

